# Therapeutic Effect of Traditional Chinese Medicine on a Rat Model of Branch Retinal Vein Occlusion

**DOI:** 10.1155/2019/9521379

**Published:** 2019-02-18

**Authors:** Pan Long, Weiming Yan, Jianwen Liu, Manhong Li, Tao Chen, Zuoming Zhang, Jing An

**Affiliations:** ^1^Center of Clinical Aerospace Medicine, Fourth Military Medical University, Xi'an, Shaanxi 710032, China; ^2^Department of Ophthalmology, The 900th Hospital of the Logistic Team of Chinese PLA, Fuzhou, Fujian 350025, China; ^3^Department of Ophthalmology of Xijing Hospital, Fourth Military Medical University, Xi'an, Shaanxi 710032, China; ^4^Institute of Neurobiology, School of Basic Medical Sciences, Xi'an Jiaotong University, Xi'an, Shaanxi 710061, China

## Abstract

Branch retinal vein occlusion (BRVO) is a common retinal vascular disorder leading to visual impairment. Currently, the general strategies for BRVO are symptomatic therapies. Cardiovascular aspects are essential risk factors for BRVO. The traditional Chinese medicine hexuemingmu (HXMM), consisting of tanshinol and baicalin, dilates the vasculature and accelerates microcirculation. Therefore, the aim of this study was to determine the efficacy and possible mechanism of HXMM in a BRVO rat model established by laser photocoagulation. Successful BRVO rat models were treated with different doses of HXMM. Fundus photography and fluorescein fundus angiography (FFA) of the animals were applied. The retinal layers were measured by optical coherence tomography (OCT). Full-field electroretinography (ffERG) was applied to evaluate the retinal function. The ear vein flow velocity was measured via a microcirculation detector. The expression of the vascular endothelial growth factor (VEGF-*α*) was measured via western blotting and immunofluorescent staining. Our study found that retinal edema predominantly occurred in the inner nuclear layer (INL) and outer nuclear layer (ONL). The retinal edema of the treated groups was significantly relieved in the early stage of BRVO as visualized via OCT detection and HE staining. The amplitudes of the *b* wave and oscillatory potentials (OPs) waves of ffERG in the treated groups were increased compared with those of the control group at several detection points (3, 5, 7, 10, 14, and 21 d postocclusion). The expression of VEGF-*α* was reduced in the treated groups at an early stage of BRVO. Furthermore, the ear vein flow velocity of the HXMM treatment groups was faster than that of the control group. Thus, our study indicates that the traditional Chinese medicine HXMM could ameliorate retinal edema and rescue the retinal structure and function in BRVO models through promoting occluded vein recanalization, improving microcirculation, and regulating the expression of VEGF-*α*.

## 1. Introduction

Retinal vein occlusion (RVO) is a common ophthalmic vessel disease that can be categorized into two types: branch retinal vein occlusion (BRVO) and central retinal vein occlusion (CRVO). BRVO is a severe human health issue with prevalence rates ranging from 0.3% to 1.1%, which is the second most frequent retinal vascular disorder [1, 2]. The prevalence rate of BRVO is five times higher than that of CRVO. Additionally, CRVO usually has a poor prognosis due to a short therapeutic window. Conversely, BRVO has a variable prognosis that depends on appropriate and effective treatments.

Currently, the general strategies for BRVO include anti-VEGF drugs against neovascularization and dexamethasone to alleviate retinal edema as first-line BRVO therapies [3, 4]. Overexpression of VEGF-*α* at the onset of BRVO is associated with complications, such as macular edema and neovascularization, mediated by the VEGF-VEGFR pathway [5]. However, VEGF-*α*, as a physical growth factor, plays an essential role in retinal protection and nutritional supply. Repressing physical VEGF-*α* is inevitable when anti-VEGF-*α* is administered to retard retinal rescue and growth. Additionally, anti-VEGF treatment is an economic burden and can lead to physical pain for patients who need persistent treatment to sustain improved visual acuity [6]. A dexamethasone implant treatment has anti-inflammatory effects and indirectly inhibits neovascularization [7]. However, high intraocular pressure, cataracts, and noninfectious ophthalmitis are the main adverse effects when used beyond the safety range [6]. Moreover, all of the above treatments are symptomatic therapies. The main etiology of BRVO is cardiovascular risk factors for the elderly, leading to BRVO with secondary damage on retinal neurons and the optic nerve.

The cardiovascular risk factors of BRVO patients are difficult to eliminate, causing irreversible visual acuity injury. There is a long history of applying traditional Chinese medicines to retinal vascular diseases. Some studies showed that traditional Chinese medicines could help reconstruct retinal vessels in an RVO model and could inhibit the neovascular factor detected by FFA [8, 9]. HXMM is a compound medicine composed of active vessel components, such as tanshinol, baicalin, and digitalis glycosides. These ingredients can inhibit platelet aggregation and adhesion, improve blood rheology, reduce the permeability of blood vessels, improve blood vessel brittleness, and prevent thrombosis [10, 11]. Therefore, traditional Chinese medicine has specific benefits for regulating the vascular microenvironment. Moreover, it is easy to apply in clinical treatment.

In this study, we applied a modified laser photocoagulation to establish a stable and repeatable BRVO rat model via a suitable laser parameter. We observed the structure of the retina and the alterations of fundus vessels and evaluated the function of the retina and fundus vessels comprehensively. Compared to the natural progression of the BRVO model, HXMM could improve the microcirculation of fundus vessels and reduce the expression of VEGF-*α* in early stages of BRVO, which ameliorated retinal edema and prevented angiogenesis during the onset of BRVO. HXMM downregulated the expression of VEGF-*α*, leading to self-healing of the fundus vessels. Therefore, the traditional Chinese medicine HXMM may provide an efficient and moderate approach for BRVO clinical therapy.

## 2. Methods

### 2.1. Animals

One hundred and ten healthy adult male Sprague Dawley (SD) rats (6 to 8 weeks old, weighing 180–220 g) were obtained from the Laboratory Animal Center of the Fourth Military Medical University in Xi'an, Shaanxi Province, China (license no. 2014270138S). All of the animals were maintained under standard laboratory conditions (room temperature of 23°C ± 3°C, 40–65% humidity, and 12 h light/dark cycles), with food and water available ad libitum. All experiments were conducted in accordance with the ARVO Statements for the use of Animals in Ophthalmic and Vision Research. Every effort was made to minimize the number of animals used and their suffering. All procedures regarding the use and handling of the animals were conducted in accordance with the Institutional Animal Care and Use Committee of the Fourth Military Medical University.

### 2.2. BRVO Model

Rats were anesthetized by intraperitoneal (IP) injection with 3 mL/kg 1% sodium pentobarbital (Sigma, St Louis, MO, USA, P3761) and 50 *μ*L sumianxin II (Jilin Shengda Animal Pharmaceutical, Co., Jilin, China). Rose bengal (50 mg/mL) (Chengdu Ai keda Chemical Reagent, Co., Sichuan, China) was injected into a tail vein, and 50 laser spots were applied to a branch vein (3 disc diameters from the optic nerve bitemporal) on the right eye of each animal using an image-guided laser system (532 nm) attached to a Micron IV Retinal Imaging Microscope (Lumenis, Inc., USA) with specific parameters (power: 80 mW; duration: 100 ms; spot size: 100 *μ*m).

### 2.3. Drug Administration

HXMM (Beilin Pharma, Xi'an, China) was stored in the dark at room temperature, and it was dissolved in normal saline at specific concentrations immediately before using. Rats that had undergone the BRVO procedure were randomly divided into 4 groups (*n* = 24). High- (HIGH-), moderate- (MOD-), and low- (LOW-) dose treatment group rats received an intragastric administration of HXMM at a dose of 16, 8, and 4 mg/kg, respectively. The control (CK) group received an intragastric administration of the same volume of normal saline. No clinical signs of toxicity in any of the HXMM-treated animals were observed during the experiment. Functional and morphological examinations were performed at time points of 1, 3, 5, 7, 10, 14, and 21 d postocclusion.

### 2.4. Histological Staining

To visualize retinal histological changes, HE staining of rat eye sections was performed at 1 d and 21 d postocclusion. Rats (*n* = 6) were euthanized and the eyes of the rats of all groups were enucleated rapidly by intraperitoneal injection of a lethal dose of sodium pentobarbital. Eyes used for histological analysis were kept immersed for at least 48 h at 4°C in a fixative solution containing 4% paraformaldehyde. Six paraffin-embedded sections (thickness: 5 *μ*m) were cut through the laser photocoagulation site according to OCT information. Eye sections were then prepared in the standard manner and stained with HE. The damage induced by retinal vein occlusion was then evaluated using three HE-stained sections from each eye for morphometric analysis. Light-microscope images were taken using a digital imaging system (DP71, Olympus, Japan), and the INL and ONL were measured at the laser photocoagulation site.

### 2.5. Immunofluorescence Staining

The immunofluorescence staining procedure was performed following the manufacturer's instructions at 1 d and 21 d postocclusion (*n* = 6). Eye sections were deparaffinized and dehydrated. Endogenous peroxidase activity was blocked by incubating the sections in 3% H_2_O_2_ for 10 min. Then, the sections were rinsed in phosphate buffer saline (PBS: 0.1 mM, pH 7.2) at room temperature 3 times every 5 minutes. After incubation with 1% bovine serum albumin (BSA) for 1 h to block nonspecific labeling, the sections were incubated overnight at 4°C with polyclonal rabbit VEGF-*α* (1 : 100; #Ab46154, Abcam, Cambridge, MA) primary antibody. Slides incubated without primary antibody served as a control. The slides were washed with PBS 3 times and incubated for 1 h with IgG (H + L) and Cy3 fluorescence secondary antibody (Zhuangzhi, EK022, Xi'an, Shaanxi province, China) at 1:200 dilution for 1 h at room temperature. After rinsing in PBS 3 times, nuclei were counterstained by incubating the sections in 100 ng/mL DAPI. Images of the slides were captured by a fluorescence microscope (BX53, Olympus, Japan).

### 2.6. Western Blot Detection

The BRVO retina tissues were separated and homogenized on ice in RIPA buffer (Beyotime, Nantong, Jiangsu, China) supplemented with 1 : 100 of the proteinases/phosphatase inhibitor (*n* = 3) at 1 d and 21 d postocclusion. Then, the lysates were centrifuged at 12,000 rpm at 4°C for 15 min to obtain the supernatant. A bicinchoninic acid (BCA) protein assay kit (Beyotime, Nantong, Jiangsu, China) was applied to determine the concentration of the protein. Equal amounts of protein were denatured by boiling in the loading sample buffer and then 30 *μ*g protein from each sample was loaded, separated by sodium dodecyl sulfate-polyacrylamide gel electrophoresis (5%, 12%). Next, the proteins were transferred onto PVDF membranes (Millipore, US) at 100 V for 120 min. The membranes were incubated with 5% nonfat milk solution for 2 h at room temperature and then reacted with VEGF-*α* (#Ab46154, Abcam, USA) at a 1 : 1000 dilution and GAPDH (Zhuangzhi Bioscience Technology Company, Xi'an, Shaanxi, China) at a 1 : 1000 dilution at 4°C overnight. The membranes were then incubated with HRP-conjugated secondary antibody (#EK020, Zhuangzhi Bioscience Technology Company, Xi'an, Shaanxi, China) at a 1 : 10000 dilution at room temperature for 1 h, and then, enhanced chemiluminescence (Thermo Fisher Scientific, Waltham, MA, US) was used for protein visualization. The intensity of immunoreactivity was quantified by densitometry using ImageJ software (NIH).

### 2.7. OCT, FFA, and Ear Microcirculation Detection

OCT images were taken at 1, 3, 5, 7, 10, 14, and 21 d after laser photocoagulation (*n* = 12), as were corresponding OCT scans using a Micron IV fundus camera and an OCT scan head equipped with a mouse objective lens. Linear OCT scans consisted of a series of 1024 single-point A-scans. Right eyes had previously been dilated with 0.5% tropicamide (Shenyang Xingji Corporation, Shenyang, Liaoning, China). Hydroxyl ethyl cellulose l (Bausch and Lomb Freda, Shandong, China) was used as a coupling gel. Fundus and OCT images were captured from 20 positions for each eye using a Retinal Imaging System (OPTO-RIS, OptoProbe, Canada) and 4D-ISOCT Microscope Imaging System (ISOCT, OptoProbe, Canada). Additionally, FFA was applied at 1, 3, 5, 7, 10, and 14 d postocclusion (*n* = 12) using HRAplusII (Heidelberg, Ger) after injecting 0.1 mL/100 g 10% fluorescence sodium (Baiyunshan Mingxing Corporation, Guangzhou, Guangdong Province, China). Ear microcirculation detection was performed at 1 d and 21 d postocclusion (*n* = 6) with a microcirculation detector (Xindi, Inc., Shanghai, China).

### 2.8. Electroretinography

Electroretinography (ERG) measurements were obtained as described previously at 0 (before BRVO), 1, 3, 5, 7, 10, 14, and 21 d postocclusion (*n* = 12). The ERG recordings were performed according to a previously described method [12]. Briefly, animals were subjected to dark adaption overnight (>12 h) and prepared for recording under a dim red light. Anesthesia was applied as described previously. The pupils were dilated with 0.5% tropicamide-phenylephrine ophthalmic solution. Corneal anesthesia was achieved with one drop of proxymetacaine (0.5%), and each cornea was kept moist with physiological saline. The pupils were dilated to a diameter of approximately 5 mm with compound tropicamide eye drops (5 mg/ml). Full-field ERGs were recorded using the full-field (Ganzfeld) stimulation and a computer system (RETI port; Roland Consult GmbH, Brandenburg, Germany) with custom-made silver chloride electrodes. The active electrode was a ring electrode placed at the center of the cornea. Stainless steel needle electrodes were placed in the cheek and tail to serve as the reference and ground leads, respectively. Dark-adapted 0.01 ERG, dark-adapted 3.0 ERG, dark-adapted oscillatory potentials, light-adapted 3.0 ERG, and light-adapted flicker ERG were recorded according to the ISCEV guidelines. Levofloxacin eye drops (Shenyang Xingji Corporation, Shenyang, Liaoning Province, China) were used three times a day after ERG testing to avoid infection.

### 2.9. Statistical Analyses

Analysis of variance (ANOVA) followed by Bonferroni's post hoc analysis was performed to examine the significant differences among all of the groups unless otherwise specified. The values are presented as the mean ± standard error of the mean (SEM), with *p* ≤ 0.05 being statistically significant.

## 3. Results

### 3.1. Establishment of the BRVO Rat Model

Fundus photography and FFA were performed to identify the established BRVO models via laser photocoagulation. The success rate of the BRVO rat model was 87.3% (96/110). The individual cases of failure included 5 deaths after laser photocoagulation, 2 spontaneous revascularizations at the first day after modeling, 4 severe exudative retinal detachments by hemorrhage, and 3 cataracts. All of the above were eliminated from the experiment.

### 3.2. Natural History

OCT and FFA were applied to observe the natural outcome of the retinal structure. The obvious obstruction of the retinal vein was found at the photocoagulation position in the BRVO rat models. The distally occluded retinal veins were distended, and the proximal vessels were beaded. OCT showed different degrees of retinal edema in the obstructed areas, including outer nuclear layer (ONL) and inner nuclear layer (INL). Additionally, adjacent no-perfusion and vasodilatation areas were observed by FFA. The occluded areas gradually reperfused at 3-4 d postocclusion in most animals.

**Figure 1:**
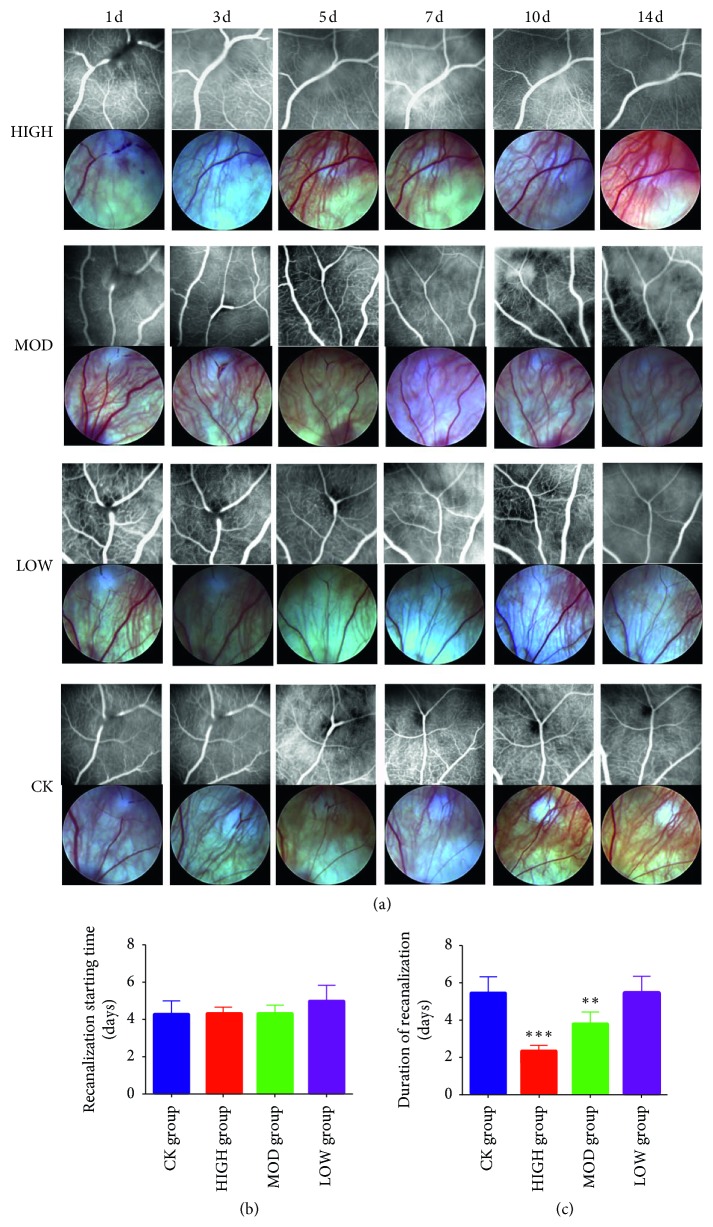
HXMM decreased occlusive vein recanalization duration and had no influence on the recanalization onset time. (a) The typical FFA and fundus photograph. (b) Recanalization starting time. (c) Duration of recanalization time. The statistical significance was determined by one-way ANOVA. Data are presented as the mean ± SEM. All analyses were performed in duplicate. *n* = 12 rats per group. ^*∗∗*^*p* < 0.01, HIGH, MOD, and LOW groups vs. CK group; ^*∗∗∗*^*p* < 0.001, HIGH, MOD, and LOW groups vs. CK group.

The onset time, duration time, and effective collateral circulation of the BRVO recanalization were detected by FFA at a series of time points. As shown in Figure 1, the time of collateral circulation establishment in the HXMM-treated groups was earlier than that of the control group. Furthermore, recanalization of the BRVO control group started at 3 d postocclusion, and the duration time was 5 d. The recanalization onset time of the HXMM-treated groups was not different compared with the control group (4.2 ± 0.8 d) (*p* > 0.05, Figure 1(b)). Moreover, there was no significant difference among the different doses of the HXMM groups (*p* > 0.05). The duration time of recanalization in the HXMM-treated groups (HIGH: 2.1 ± 0.3 d; MOD: 3.9 ± 0.4 d; LOW: 5.7 ± 0.8 d) was less than the control group (5.4 ± 0.8 d) in a concentration-dependent manner (all *p* < 0.05, Figure 1(c)).

**Figure 2:**
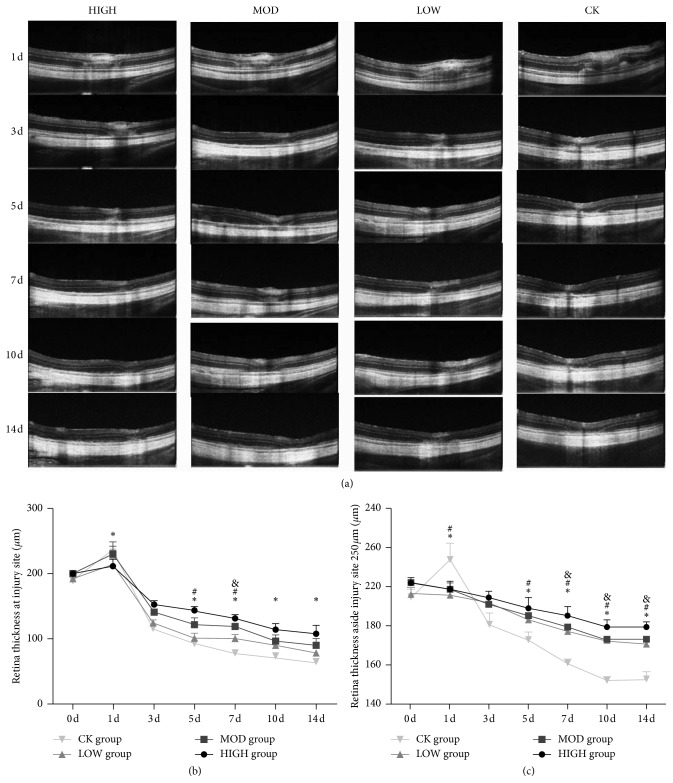
HXMM alleviated BRVO rat retinal edema and protected BRVO rat retinal integrity. (a) The typical OCT pictures of injured BRVO rat retina treated with different doses of HXMM at 1, 3, 5, 7, 10, and 14d. (b, c) The thickness of the BRVO rat retina treated with different doses of HXMM at the injury site and 250 *µ*m from the injury site measured by OCT. The statistical significance was determined by one-way ANOVA. Data are presented as the mean ± SEM. All analyses were performed in duplicate. *n* = 12 rats per group. ^*∗*^*p* < 0.05, HIGH group vs. CK group; ^#^*p* < 0.05, MOD group vs. CK group; ^&^*p* < 0.05, LOW group vs. CK group.

OCT was performed to monitor the changes in retinal thickness. As shown in Figure 2, at 1 d postocclusion, retinal edema occurred predominantly in the inner retinal layers, especially in the inner plexiform layer (IPL), and the outer nuclear layer (ONL) was increased and disordered. Furthermore, the thicknesses of the retina in HXMM-treated groups were thinner than that of the control group (*p* < 0.05). The retinal edema gradually absorbed from 3 d postocclusion, and the thickness of retinal layers almost returned to the level before BRVO. Interestingly, 5 days after BRVO, the entire retinal thickness, including the ganglion cell layer (GCL) and ONL, was dramatically decreased, especially the ONL layer. The thickness of the retina in the HXMM-treated group was thicker than that of the control group (*p* < 0.05, Figure 2(a)). A distance approximately 250 *µ*m from the laser photocoagulation vein was the photocoagulation area. The thickness in the HIGH HXMM-treated group was greater compared with those of the medium- and low-dose groups and control group (*p* < 0.05, Figure 2(c)). The protective effects, including alleviating retinal edema and protecting retinal structure integrity, were partly observed in a concentration-dependent manner.

**Figure 3:**
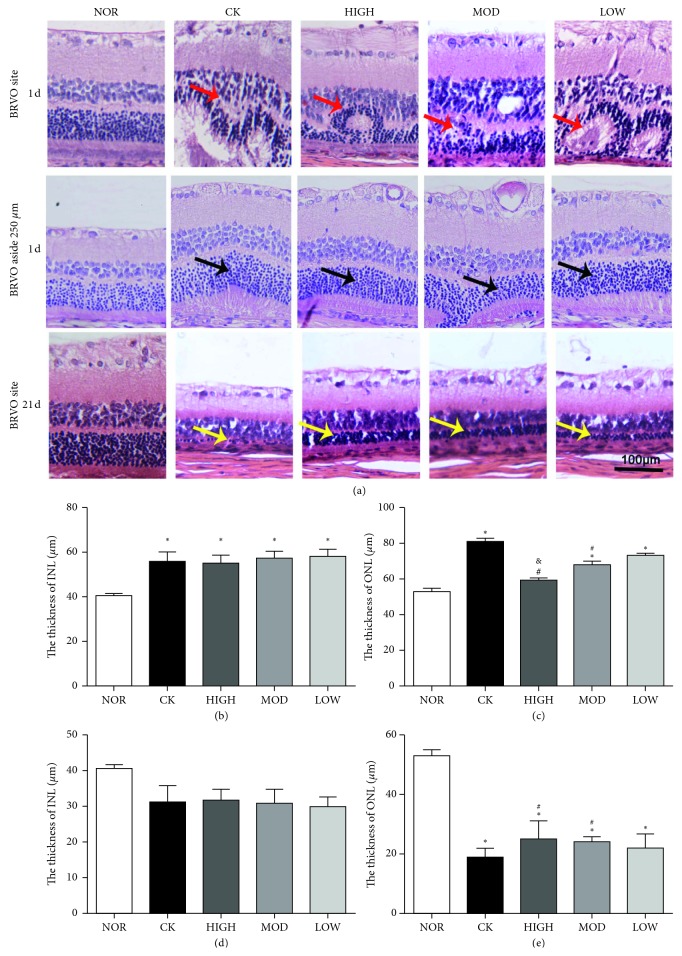
HXMM alleviated BRVO rat retinal edema and protected BRVO rat retinal integrity. (a) The typical HE pictures of normal and BRVO rat retina treated with different doses of HXMM at 1 d and 21 d postocclusion. (b, c) The thickness of INL and ONL of normal and injured BRVO rat retina treated with different doses of HXMM at 1 d postocclusion. (d, e) The thickness of INL and ONL of normal and BRVO rat retina treated with different doses of HXMM at 21 d postocclusion. The red arrow denotes retina disorder; the black arrow denotes ONL edema; and the yellow arrow denotes a thinner ONL. The statistical significance was determined by one-way ANOVA. Data are presented as the mean ± SEM. *n* = 6 rats per group. ^*∗*^*p* < 0.05, HIGH, MOD, LOW, and CK groups vs. NOR group; ^#^*p* < 0.05, HIGH, MOD, and LOW groups vs. CK group; ^&^*p* < 0.05, HIGH and MOD groups vs. LOW group.

H&E staining showed changes in the retinal structure with progressing stages of BRVO. The thickness of ONL and INL was calculated at 1 d (peripheral area and photocoagulation area) and 21 d postocclusion (photocoagulation area). As shown in Figure 3, the thickness of the INL and ONL was remarkably increased at 1 d while significantly decreased at 21 d postocclusion in the control group (1 d: ONL, 81 ± 2 *μ*m, INL, 51.25 ± 4.57 *μ*m; 21 d: ONL, 19 ± 2.94 *μ*m, INL, 30.11 ± 6.18 *μ*m) compared with those of the nonoperation group (ONL, 53 ± 2 *μ*m, INL, 39.25 ± 4.57 *μ*m). Meanwhile, we could find that photocoagulation site retina, including INL and ONL, was totally disordered at 1 d postocclusion. Although the HXMM-treated groups showed no significant difference in the thickness of INL at both 1 d and 21 d postocclusion compared with that of the control group (*p* > 0.05, Figures 3(b) and 3(d)), the thickness of the ONL in the HXMM-treated groups was thinner at 1 d and thicker at 21 d postocclusion than that of the control group (*p* < 0.05, Figures 3(c) and 3(e)).

### 3.3. Retinal Function of the BRVO Rat Model after Treatment

**Figure 4:**
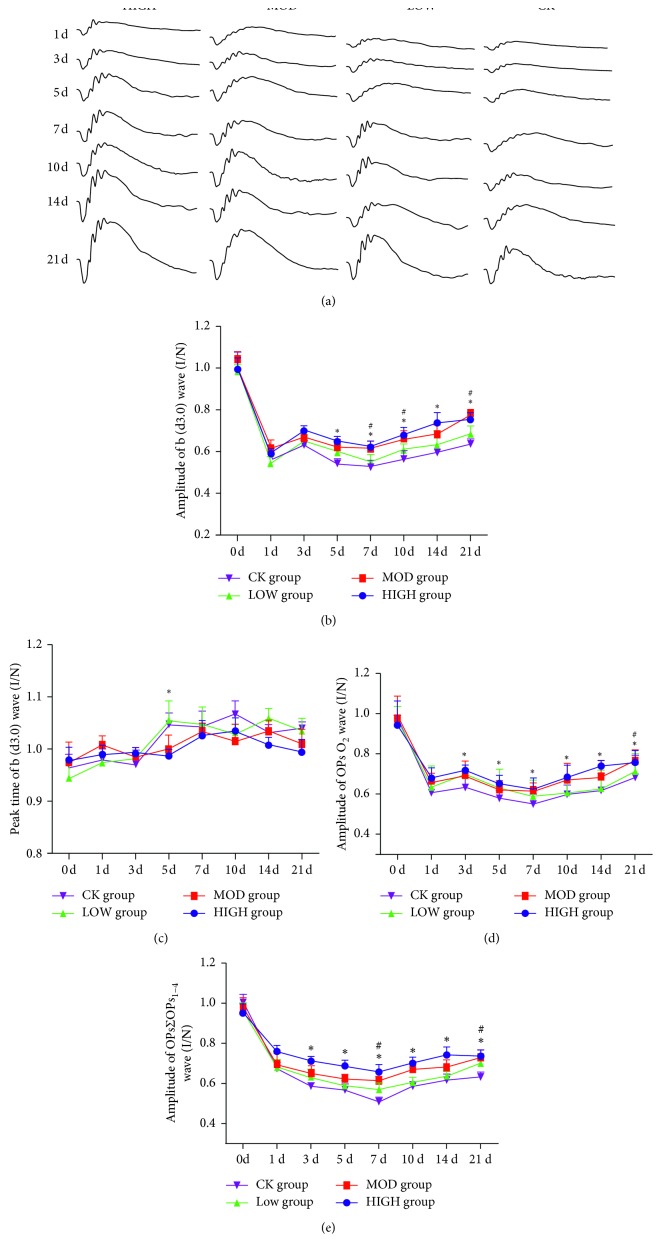
HXMM improved BRVO rat retinal function. (a) The typical ERG (dark-adaptation response 3.0) performance. (b) BRVO rat amplification of the *b* (d3.0) wave after modeling 0, 1, 3, 5, 7, 10, 14, and 21 d postocclusion. (c) BRVO rats peak time of the *b* (d3.0) wave after modeling 0, 1, 3, 5, 7, 10, 14, and 21 d postocclusion. (d) BRVO rat amplitude of the OPsO_2_ wave after modeling 0, 1, 3, 5, 7, 10, 14, and 21 d postocclusion. (e) BRVO rat amplitude of the ∑OPs_1–4_ wave after modeling 0, 1, 3, 5, 7, 10, 14, and 21 d postocclusion. The statistical significance was determined by one-way ANOVA. Data are presented as the mean ± SEM. All analyses were performed in duplicate. *n* = 12 rats per group. ^*∗*^*p* < 0.05, HIGH group vs. CK group; ^#^*p* < 0.05, MOD group vs. CK group.

Retinal function was measured using ffERG to assess the electrical activity of retinal cells. The amplitudes of *a* and *b* wave reduction are associated with macular edema and retinal thickness in patients with retinal vein occlusion [13, 14]. Twelve rats in each group as previously described underwent ERG tests at 1, 3, 5, 7, 10, 14, and 21 d after BRVO. The amplitudes of the *b* wave of the dark-adaptation 3.0 response and OPs response were significantly decreased to 50–60% of baseline at the onset of BRVO (Figure 4(a)). On the 3rd day, the photocoagulated vein began to recanalize. Simultaneously, obvious improvements could be recognized in both the dark-adaptation 3.0 response and OPs response of the HXMM-treated groups (d3.0 : 306.98 ± 109.15 *μ*V; OPsO_2_: 175.34 ± 54.78 *μ*V) compared with those of the control group (d3.0 : 236.37 ± 57.05 *μ*V; OPsO_2_: 111.77 ± 32.53 *μ*V) (*p* < 0.05, Figure 4(b)). However, at 5 d postocclusion, the amplitudes of the *b* wave of the dark-adaptation 3.0 response and OPs response decreased until 7 d after BRVO. Interestingly, the *b* wave of the dark-adaptation 3.0 response and OPs response increased 7 d after BRVO, reaching 60–70% of baseline (d3.0 : 692.45 ± 135.82 *μ*V; OPsO_2_: 267.33 ± 72.88 *μ*V). Unlike the former observation, the amplification of the ∑OPs_1–4_ wave decreased the entire time until 7 d after BRVO and then increased slightly to 60–65% of baseline. The HXMM-treated groups had lower decreased amplitudes of the *b* wave of the dark-adaptation 3.0 response and OPsO_2_ response compared with the control group (*p* < 0.05). Regarding the amplitudes of the ∑OPs_1–4_ wave, the HXMM-treated groups had an increasing value compared with the control group (*p* < 0.05). The peak time of the dark-adaptation 3.0 response *b* wave in the HXMM-treated groups (61.22 ± 5.54 ms) was less than the control group (71.13 ± 4.47 ms) (*p* < 0.05, Figure 4(c)). In addition, the *b* wave amplitudes of the dark-adaptation 3.0 response, OPsO_2_ wave, and ∑OPs_1–4_ wave were partly different among each dose in the HXMM groups (*p* < 0.05, Figures 4(d) and 4(e)).

### 3.4. Microcirculation of the Ear Vein in the BRVO Rat Model after Treatment

**Figure 5:**
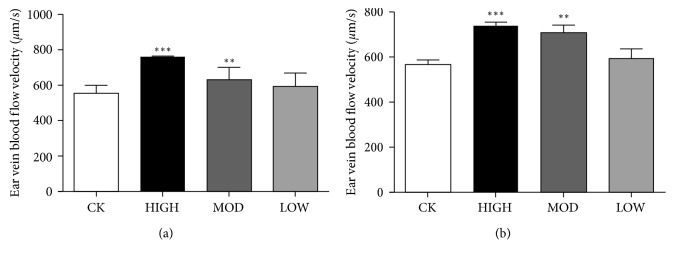
The effect of HXMM on BRVO rat microcirculation. BRVO rat ear vein blood flow velocity at 1 d (a) and 21 d (b). The statistical significance was determined by one-way ANOVA. Data are presented as the mean ± SEM. All analyses were performed in duplicate. *n* = 6 rats per group. ^*∗∗*^*p* < 0.01, HIGH, MOD, and LOW groups vs. CK group; ^*∗∗∗*^*p* < 0.001, HIGH, MOD, and LOW groups vs. CK group.

Microcirculation is an important indicator of prognosis in vascular disease. In this study, a microcirculation detector was applied to the BRVO models with or without HXMM treatment. The results showed that the flow velocity of ear vein blood in the HXMM-treated groups (1 d: 758 ± 59 mm/s; 21 d: 729 ± 34 mm/s) was increased at 1 d and 21 d after the BRVO operation compared with the control group (1 d: 556 ± 44 mm/s, 21 d: 567 ± 41 mm/s) (*p* < 0.05, Figures 5(a) and 5(b)). However, the diameter of the ear vein showed no significant difference among the groups (*p* > 0.05).

### 3.5. The Expression of VEGF-*α* in the BRVO Rat Model after Treatment

**Figure 6:**
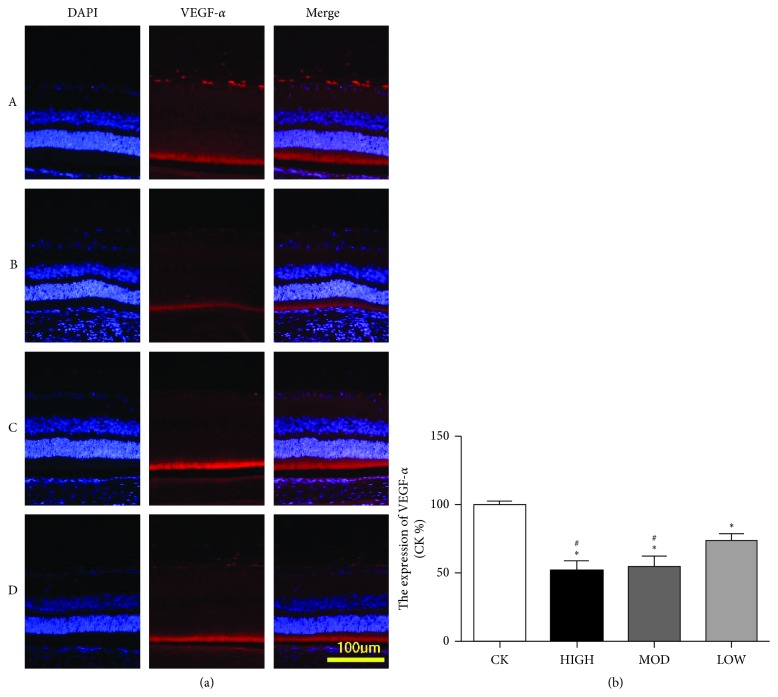
HXMM decreased the expression of VEGF-*α* at 1 d postocclusion. (a) The typical immunofluorescence staining pictures of the BRVO rat retina with or without HXMM treatment at 1 d postocclusion. (b) The expression of VEGF-*α*: CK group, HIGH group, MOD group, and LOW group. The statistical significance was determined by one-way ANOVA. Data are presented as the mean ± SEM. All analyses were performed in duplicate. *n* = 6 rats per group. ^*∗*^*p* < 0.05, HIGH, MOD, and LOW groups vs. CK group; ^#^*p* < 0.05, HIGH and MOD groups vs. LOW group.

**Figure 7:**
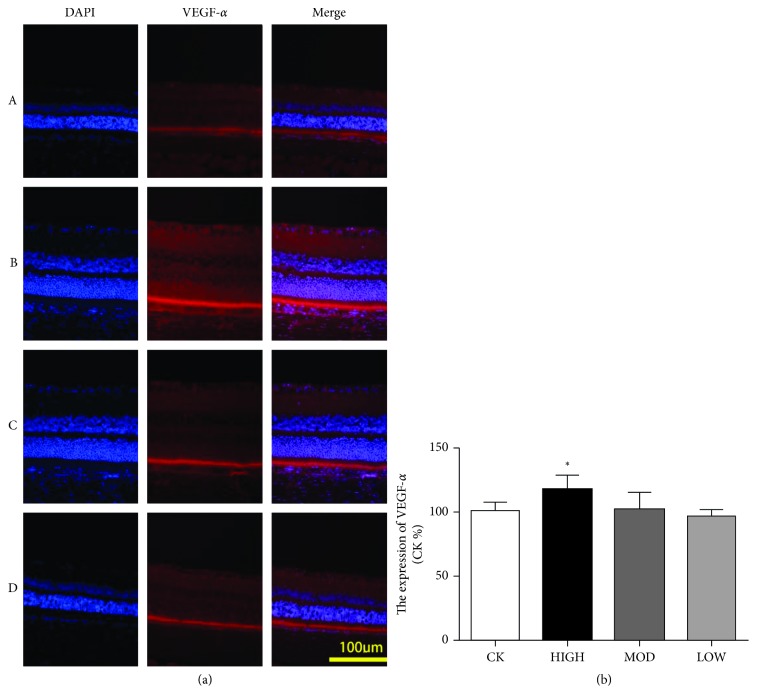
HXMM slightly increased the expression of VEGF-*α* at 21 d postocclusion. (a) The typical immunofluorescence staining pictures of the BRVO rat retina with or without HXMM treatment at 21 d postocclusion. (b) The expression of VEGF-*α*: CK group, HIGH group, MOD group, and LOW group. The statistical significance was determined by one-way ANOVA. Data are presented as the mean ± SEM. All analyses were performed in duplicate. *n* = 6 rats per group. ^*∗*^*p* < 0.05, HIGH, MOD, and LOW groups vs. CK group.

**Figure 8:**
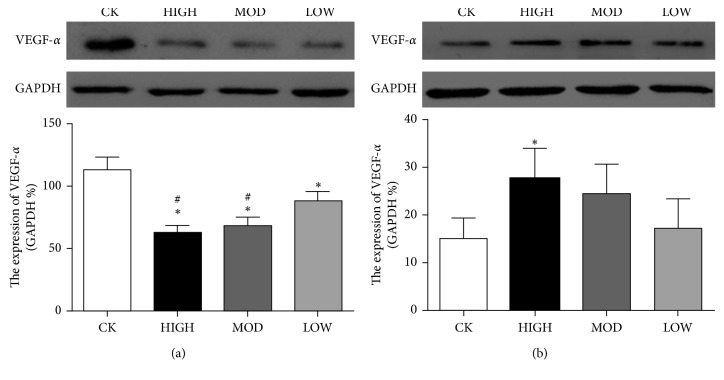
The expression of VEGF-*α* in BRVO retina at 1 d (a) and 21 d (b) postocclusion. *n* = 6 rats per group, mean ± SEM; ^∗^*p* < 0.05, HIGH, MOD, and LOW groups vs. CK group; ^#^*p* < 0.05, HIGH and MOD groups vs. LOW group.

To explore the therapeutic effect of HXMM on the expression of retinal VEGF-*α* after BRVO, immunofluorescence and western blotting were performed. The immunofluorescence results showed that the expression of VEGF-*α* in the inner retina layer was decreased in the HXMM-treated groups compared with that of the control group 1 d after BRVO (*p* < 0.05). HXMM inhibited the expression of VEGF-*α* in a concentration-dependent manner (*p* < 0.05, Figure 6). Furthermore, the high dose of HXMM slightly increased the expression of VEGF-*α* at 21 d postocclusion (*p* < 0.05, Figure 7). The western blot result was consistent with the immunofluorescence (Figure 8).

## 4. Discussion

BRVO is a common ophthalmological disease that severely damages visual acuity with an increasing prevalence related to age and represents a burden to society. To explore BRVO progression and changes in retinal structure and function, a suitable BRVO animal model is urgently needed. There are several methods to establish BRVO animal models, including surgical intervention, thrombin intravitreal injection, and laser photocoagulation [15–17]. Early laser photocoagulation methods did not mimic retinal edema or the retinal nonperfusion area [18]. In this study, we used a modified laser photocoagulation. First, we selected a suitably sized vein as a target to avoid severe damage and to ensure rapid recanalization of the vein. Secondly, 80 mW, 100 ms, 100 *µ*m, and 50 spots were chosen as the proper parameters according to preliminary experiments. Half of the spots were performed first with a 10 s waiting period followed by the other half of the spots to better obstruct the target vein. Importantly, good cooperation between the operators led to a higher success rate.

The inner retinal layers, especially the INL and GCL, are affected after suffering BRVO [15, 19]. OCT measurements and H&E staining were performed to observe the retinal layer in vivo and in vitro, respectively. The results showed that HXMM could effectively alleviate edema in the inner layer, especially the INL and retinal nerve fiber layer (RNFL) in the early (1 d postocclusion) stages. However, our results also showed that the ONL was notably affected in the untreated control group throughout the progression of BRVO. It is hypothesized that photoreceptor cells are sensitive to hypoxic-ischemic damages [20], leading to the exudation of ONL in the early stage and the attenuation in the later stage of BRVO. Compared with the natural progression of BRVO in the control group, HXMM played essential roles in protecting INL cells via alleviating retinal edema in the early stages and reducing retinal cell death of ONL in the later stages. The flow velocity of ear vein blood was increased at the onset of BRVO in treated groups compared with that of the untreated group. We speculated that the active ingredients tanshinol and baicalin [8, 9] of HXMM could improve retinal microcirculation and promote recanalization via sustaining a better retinal microenvironment. Studies have found that tanshinol could attenuate fibrosis oxidative stress by nuclear factor erythroid 2-related factor 2/heme oxygenase (Nrf2/HO-1) signaling pathways [21]. Furthermore, baicalin could ameliorate high glucose-induced vascular inflammation and improve vascular endothelial function via decreasing inflammatory damage and oxidative stress [22–25]. Additionally, the roles of the antithrombotic and profibrinolytic activities of baicalin have been confirmed by numerous studies [26]. In the study, we found HXMM could protect retinal photoreceptors and promote edema absorption of the retina during the progression of BRVO.

Retinal function in the HXMM treated groups was restored both in rod and cone mixed responses and blood vessel functions compared with those of the untreated group. Intriguingly, there was a rapid decline of the ffERG responses from 3 d to 5 d after occlusion in all of the groups. The declining ERG responses may be associated with ischemia reperfusion [27]. HXMM could rescue the retinal function and protect the function of retinal blood vessels.

Currently, anti-VEGF-*α* therapy is an effective intervention for BRVO disease. However, the effect of the VEGF treatment is associated with underlying disease, occlusive sites, intervention time and interval time, and individual specificity [28, 29]. Moreover, many individuals do not suffer from obvious side effects [28, 30, 31]. The reason for the different efficacies of VEGF treatment remains unclear. VEGF-*α* plays different roles in different stages, which are harmful for neovascular growth at disease onset and beneficial for self-healing in the later periods [32, 33]. In this study, the expression of VEGF-*α* in BRVO treated rats was reduced at the early stages of BRVO (at 1 d postocclusion), when VEGF-*α* was considered to impede angiogenesis at the early stages of BRVO. Conversely, the expression of VEGF-*α* in the treated group was increased compared with that of the untreated group at the later stages of BRVO. HXMM could upregulate VEGF-*α* to reconstruct collateral circulation and recanalize occluded retinal blood vessels. One of HXMM's active components, tanshinol, can inhibit VEGF secretion and hypoxia inducible factor 1*α* (HIF-1*α*) expression under hypoxic conditions [34, 35]. When BRVO patients were treated with anti-VEGF therapy, VEGF suppression had adverse effects on retina repair in the later stages of BRVO. Our results presented that the traditional Chinese medicine compound HXMM could regulate the expression of VEGF to control angiogenesis and recanalization at different stages of BRVO.

In conclusion, we successfully established a BRVO model to research the efficacy and mechanism of the BRVO disease with HXMM treatment. HXMM treatment presented an efficient outcome by alleviating retinal edema, accelerating retinal microcirculation, protecting visual function, and regulating VEGF-*α* expression throughout disease progression. In summary, the study provided theoretical support that the Chinese traditional medicine HXMM is a beneficial clinical treatment for BRVO disease.

## Data Availability

The original data used to support the findings of this study are available from the corresponding author upon request.
